# Salivary Calculus

**Published:** 1885-02

**Authors:** B. M. Conlin


### Salivary Calculus.

BY B. M. CONLIN, D. D. S.

Salivary calculus, commonly called tartar, is a deposit of a calcarious material occurring on the teeth, which generally shows a tendency to collect on certain teeth, so located in the mouth, that they seem to favor its retention.

In the economy of nature it has been provided that certain nutritive principles, which go to make up the various organs, which are necessary to the proper performance of vital functions, shall be constantly supplied with material to repair the waste which their activity involves.

As the osseous system, which preserves the proper form of the body and also for protecting the more delicate structures beneath, is made up largely of the various salts of lime, it is necessary that nature should make some provision for the repair of the constant waste, which these organs, like all others of the body, are constantly undergoing. Thus we find it necessary to partake of those articles of food which yield those principles of nutrition. In nature's laboratory are wrought those wonderful changes, by which those salts, which are held in solution in the serum of the blood, are converted into bone tissue. Not only are the mineral salts found in the blood, but other of the animal fluids contain them, and, indeed, they are found in nearly every tissue of the body in greater or less quantity. Thus in the bile, we often find biliary calculi deposited in the gall-bladder, giving rise to the most excrutrating pain as the smaller of the concretions pass down the duct of the gall-bladder. They have even been found in the substances of the liver and pancreas. In the urine we have calculi giving rise to stone in the bladder.

But what most concerns our present purpose, is the consideration of the precipitation, which occurs from the saliva. The saliva is the product of the secretion of three pairs of glands and the mucous membrane of the mouth. Analysis of saliva varies somewhat in the proportion of its ingredients, which are generally the following: Water, sodium chloride, ptyalin, fat, potassium chloride, calcium phosphate, and sulpho-cyanide of potassium. Normal saliva is alkaline in reaction, but the mucous is acid. The function of the saliva is to lubricate the mouth, assist in deglutition and convert hydrated starch into grape sugar by means of its active principle, ptyalin. On boiling its action is permanently altered, and temporarily suspended at the freezing point.

Tartar, the term commonly applied to this precipitate from the saliva, varies in color, consistency, and constitution in different individuals. It also varies somewhat with the age of the person, being more dense in the adult. In color it varies from a black to a hue even lighter than the tooth upon which it adheres; in consistency, from a pasty mass to a density nearly equal to the teeth, that formed most rapidly, being softer, and *vice versa*; and in constitution it varies greatly, depending on the general constitution of the individual.

It is not found in all mouths, but generally is, and in some much more than in others. It must, then, be taken that its deposit is due to some pathological condition, but just what that condition is, it is difficult to determine. Observations seem to indicate that it is most abundantly precipitated when there is an abundant alkaline, sluggish secretion. The acid mucous secretion has been charged with producing this precipitate. Carbon dioxide, exhaled from the lungs, might cause a precipitate of calcium carbonate. When the saliva is heavily loaded with the calcarious salts, and held feebly in solution, the lowering of the temperature on being ejected into the mouth, would tend to cause precipitation, as heat favors solution.

Thus in persons who habitually breath through the mouth, we always find a greater amount of deposit on the teeth than in persons who inhale and exhale through the proper organs.

It is more abundantly precipitated in adults than in young persons. This, perhaps, can be accounted for on the hypothesis that during the building up period of life, nature can appropriate all mineral material ordinarily furnished, and that in adult life, more is furnished than can be assimilated, and thus it is expelled from the system. But this might be objected to from the fact that the saliva is not an excrementitious fluid, but one designed to carry out physiological functions. At all events, older persons are more subject to this deposit.

That it is precipitated soon after coming into the mouth seems evident from the fact that largest accumulations are found nearest the orifices of the salivary ducts.

There are cases in which the calcarious salts are held so loosely in solution that precipitation takes place before passing through the duct, thus giving rise to calculi within the duct, and even in the substance of the gland itself.

These cases are rare, and to the careless observer, obscure in their diagnosis. Sometimes such deposit has been mistaken for a tumor. Accumulating in the duct it will obstruct the flow of saliva, causing severe pain in the region of the gland.

Salivary calculus is found most abundantly on the buccal surface of the first and second molars, opposite the orifice of Steno's duct, and on the lingual surfaces of the anterior, inferior teeth.

The deposit begins about the necks of the teeth, beneath the free margin of the gums, and a nucleus once established, the deposit may go on indefinitely. If no provision is made for its removal, the accumulation will continue until the crowns are entirely imbeded, and it may even cover the soft parts adjoining the teeth.

Nothing can exceed the unsightly appearance thus presented, and it is a wonder how anybody, living in this æsthetic age and having any regard for personal appearance, can tolerate it.

Even those who are most scrupulous in looking to the proper care of the rest of the body, and who would not, for anything, be considered uncleanly or untidy about their toilet, are wholly negligent on this very important point.

As a well-kept denture is one of the most beautiful things that can adorn the human features, so is an ill-kept one the most unsightly thing to behold.

All deposits that discolor the teeth are not salivary calculus, but are supposed to be deposited from the secretions of the mucous glands, mingled with the debris of the oral cavity.

It exercises no injurious effect upon the tooth structure itself; indeed, cases are known where a deposit has stopped the action of caries, but indirectly its effects are most injurious. Its adhesion near the free margin of the gum causes a rough surface which constantly irritates the mucous membrane, causing it to become swollen, congested, and consequently, very much diseased, so that a dentist can tell at a glance whether there is an accumulation or not. So long as the irritant remains, there will be an unhealthy condition of the mouth. It will go so far as to even cause suppuration of the membrane about the roots of the teeth, vitiating the secretions of the mouth which are destined to pass into the stomach. It does not require much physiology to understand what a deleterious effect this will have on the system. The breath also will become contaminated.

But it works a still greater evil in another way. Insinuating itself between the gum and tooth, it constantly encroaches upon and diminishes the periostial supply, and eventually lifting the teeth from their sockets. Whole dentures have thus been destroyed, and yet people wonder why their teeth fail.

Prof. Garretson relates a case of a woman who received an injury affecting the sub-maxillary gland, which produced ranula and terminating with a calcarious deposit within the gland, giving the patient terrible pain at times. After each paroxysm, the tumor was noticed to have increased in size, and there would be a discharge of a pasty substance resembling a " whitish worm," to use the patient's own expression. The case had been pronounced cancer by several physicians, who refused to operate, and declared that any attempt at its removal would be her death warrant.

After much persuasion it was ultimately removed, and proved to be a calcarious deposit within the gland.

Many cases resembling the above may be found in dental literature relating to this subject, but it is deemed superfluous to introduce them here.

*Treatment.*—The treatment may be local and constitutional. By constitutional treatment such means should be employed as shall correct any predisposing cause to its deposit, such as defective nutrition.

But by far the greater part will consist of local treatment, and this depends very much upon the patient himself. So far as the dentist is concerned, the local treatment consists in removing the deposit, and giving such instructions to the patient as shall enable him to keep the teeth free from accumulations in the future.

Its removal is effected in various ways by different operators; some removing by means of suitable instruments, while others prefer a dilute acid. We think much objection can be offered to the latter mode, as the composition of the deposit resembles so much that of the teeth, that what will dissolve the deposit, will, in all probability, attack and dissolve the enamel, a circumstance to be much deprecated, as it leaves it in a roughened condition, facilitating decay. We should much prefer the use of instruments, and we think this view is endorsed by the best operators.

Its removal is easily accomplished by the skillful use of properly formed instruments. These should be of various shapes and proper curvature. The blade of the instrument is passed beyond the deposit, next the gum, and by a gentle sweep of the instrument, it is easily scaled off. Care should be taken not to roughen or abrade the enamel. It often may require several sittings before all is removed. After this has been accomplished, the enamel should be smoothly polished and burnished. Pummice stone on a wooden point is a very good thing for polishing. Too much care can not be exercised in cautioning the patient against neglect, and the constant care required to keep the tartar from accumulating.

The frequent use of a proper brush and powder will generally accomplish this result, but no powder should be used that will scratch the enamel, or that contains any ingredients insoluble in the secretions of the mouth, as they may produce irritation by lodging beneath the free margin of the gum.

